# Recent advances in understanding idiopathic pulmonary fibrosis

**DOI:** 10.12688/f1000research.8209.1

**Published:** 2016-05-31

**Authors:** Cécile Daccord, Toby M. Maher

**Affiliations:** 1 Interstitial Lung Disease Unit, Royal Brompton and Harefield NHS Foundation Trust, London, UK; 2 Respiratory Medicine Department, Lausanne University Hospital, Lausanne, Switzerland; 3 NIHR Respiratory Biomedical Research Unit, Royal Brompton Hospital, London, UK; 4 Fibrosis Research Group, Imperial College, London, UK

**Keywords:** idiopathic pulmonary fibrosis, interstitial lung diseases

## Abstract

Despite major research efforts leading to the recent approval of pirfenidone and nintedanib, the dismal prognosis of idiopathic pulmonary fibrosis (IPF) remains unchanged. The elaboration of international diagnostic criteria and disease stratification models based on clinical, physiological, radiological, and histopathological features has improved the accuracy of IPF diagnosis and prediction of mortality risk. Nevertheless, given the marked heterogeneity in clinical phenotype and the considerable overlap of IPF with other fibrotic interstitial lung diseases (ILDs), about 10% of cases of pulmonary fibrosis remain unclassifiable. Moreover, currently available tools fail to detect early IPF, predict the highly variable course of the disease, and assess response to antifibrotic drugs.

Recent advances in understanding the multiple interrelated pathogenic pathways underlying IPF have identified various molecular phenotypes resulting from complex interactions among genetic, epigenetic, transcriptional, post-transcriptional, metabolic, and environmental factors. These different disease endotypes appear to confer variable susceptibility to the condition, differing risks of rapid progression, and, possibly, altered responses to therapy. The development and validation of diagnostic and prognostic biomarkers are necessary to enable a more precise and earlier diagnosis of IPF and to improve prediction of future disease behaviour. The availability of approved antifibrotic therapies together with potential new drugs currently under evaluation also highlights the need for biomarkers able to predict and assess treatment responsiveness, thereby allowing individualised treatment based on risk of progression and drug response. This approach of disease stratification and personalised medicine is already used in the routine management of many cancers and provides a potential road map for guiding clinical care in IPF.

## Introduction

Idiopathic pulmonary fibrosis (IPF) is typically introduced as a chronic progressive and inevitably fatal scarring lung disease with a prognosis worse than that of numerous cancers ^1, 2^. Hopefully, this is now beginning to change. Although the etiology and the pathogenesis of IPF are still incompletely understood, two antifibrotic drugs, pirfenidone and nintedanib, have recently been proven to be effective in slowing disease progression and are now approved as treatments in the United States and Europe ^3, 4^.

The recent development of affordable, high-throughput -omics technologies has opened the era of systems biology and has enabled the emergence of stratified and personalised medicine. These approaches are becoming routine practice in oncology ^5^ and have enormous potential in offering new insights into the understanding and management of pulmonary diseases ^6^, including IPF.

This article aims to provide an overview of recent developments in disentangling the complex interrelated mechanisms involved in the pathogenesis of IPF with a particular focus on those that may lead to improved diagnosis, stratification of disease behaviour, and identification of potential novel therapeutic targets and predictors of response to treatment. Considerations concerning the past, present, and future pharmacotherapy of IPF were addressed in the March 2014 issue of this journal ^7^ and will not be discussed in this current review.

## Diagnosis

**Figure 1.**
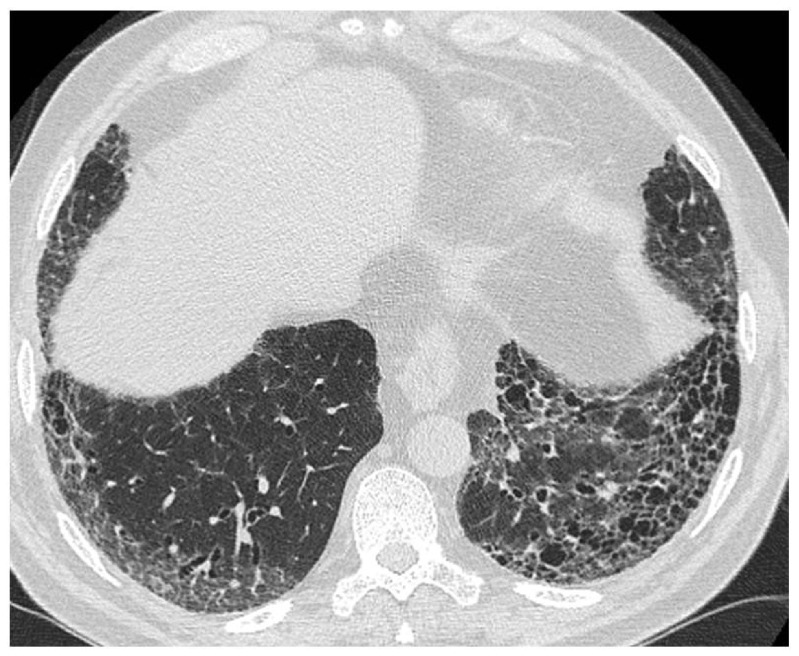
Typical high-resolution computed tomography (HRCT) pattern of usual interstitial pneumonia (UIP). The image shows subpleural and basal predominance of reticular opacities associated with traction bronchiectasis and honeycomb change (clustered cystic airspaces with well-defined thick walls and diameter of 0.3–1.0 cm).

**Figure 2.**
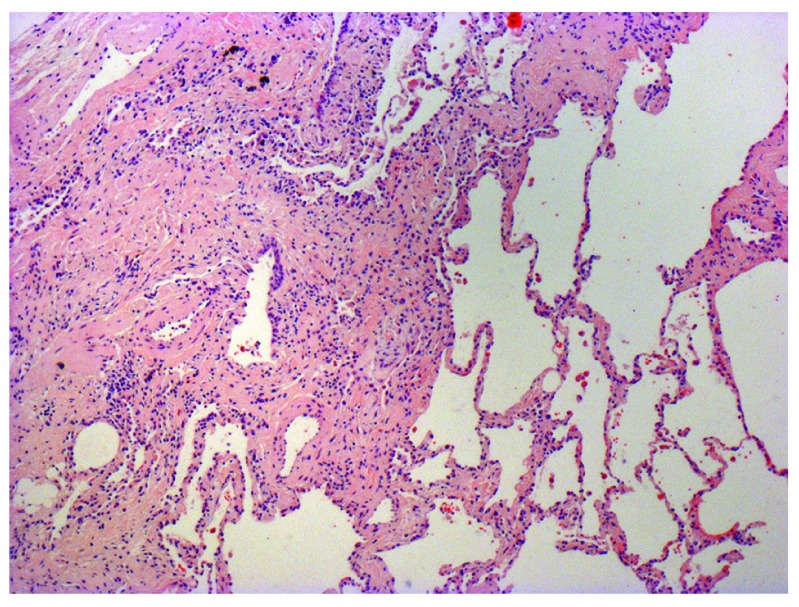
Photomicrograph of biopsy from a 63-year-old man with a multi-disciplinary diagnosis of idiopathic pulmonary fibrosis. The patient shows the typical histopathological features of usual interstitial pneumonia characterised by spatial heterogeneity with areas of subpleural and paraseptal fibrosis and honeycombing changes (cystic airspaces lined by bronchiolar epithelium) alternating with areas of relatively spared lung parenchyma, temporal heterogeneity with admixed areas of active fibrosis with fibroblast foci, extracellular matrix deposition (mainly collagen), and relative mild or absence of inflammatory cell infiltrate together with regions of histologically normal lung tissue.

The current approach to IPF diagnosis was first described in international guidelines published in 2001, which were recently updated. These guidelines define precise diagnostic criteria based on clinical, radiological, and histopathological features ^8^ and enshrine the place of multidisciplinary discussion among experienced clinicians, radiologists, and pathologists as the gold standard method for establishing a diagnosis of IPF. Using the current guidelines, in about two-thirds of the cases, a confident diagnosis of IPF can be achieved based on an appropriate clinical history in association with a typical high-resolution computed tomography (HRCT) pattern of usual interstitial pneumonia (UIP) ( Figure 1). When clinical and HRCT data are non-diagnostic, surgical lung biopsy (SLB) is recommended to confirm UIP diagnosis histologically ( Figure 2). However, SLB carries considerable risks and is often contraindicated in older patients with extensive co-morbidities or in those presenting with advanced lung disease ^9^. Thus, even in experienced centres, a diagnosis of unclassifiable interstitial lung disease (ILD) is assigned to about 10% of patients who present with progressive pulmonary fibrosis ^10^.

In a recent study of 117 patients with fibrotic ILDs, bronchoscopic lung cryobiopsy has proven to be safe and effective in providing adequate lung tissue samples, which enabled increased diagnostic confidence in the multidisciplinary diagnosis of IPF ^11^. This minimally invasive technique represents an attractive alternative to SLB and may, pending further studies, be included in the diagnostic algorithm of IPF and other fibrotic ILDs in the near future.

## Pathogenesis

The heterogeneity in radiological and histopathological appearances, rate of progression, and treatment response observed in individuals with IPF suggests that fibrosis arises as a consequence of multiple co-activated pathogenic pathways, all of which are influenced by complex interactions between endogenous and environmental factors ^12^. This multiple-pathway model probably explains the disappointing results of therapies targeting single receptors or pathways in IPF. Future treatment strategies in IPF are likely to focus on combinations of therapies targeting multiple pathogenic pathways simultaneously, as is currently used in the treatment of many cancers ^13^.

Until 15 years ago, the prevailing pathogenic paradigm in IPF was one of chronic inflammation being the precursor to progressive fibrosis. This has shifted over the last decade to a model of abnormal wound healing response driven by persistent or recurrent alveolar epithelial microinjuries (e.g. cigarette smoke, microaspiration, or infection) in individuals rendered susceptible by ageing or genetic predisposition ^14^. Multiple studies have shown that alveolar epithelial cell (AEC) apoptosis secondary to injury is followed by extravascular coagulation, immune system activation, and aberrant persistent activation of AECs, even in the absence of the primary stimulus ^15^. These cells, in turn, induce the migration and proliferation of local fibroblasts, recruit circulating fibrocytes to areas of injury, and promote differentiation of fibroblasts into myofibroblasts. This results in the formation of myofibroblast foci, the histologic hallmark of UIP, in which persistently activated myofibroblasts secrete excessive amounts of extracellular matrix (ECM) proteins. Disordered deposition and accumulation of ECM components within the interstitium and alveolar spaces lead to established fibrosis with progressive destruction of lung architecture and loss of function.

**Figure 3.**
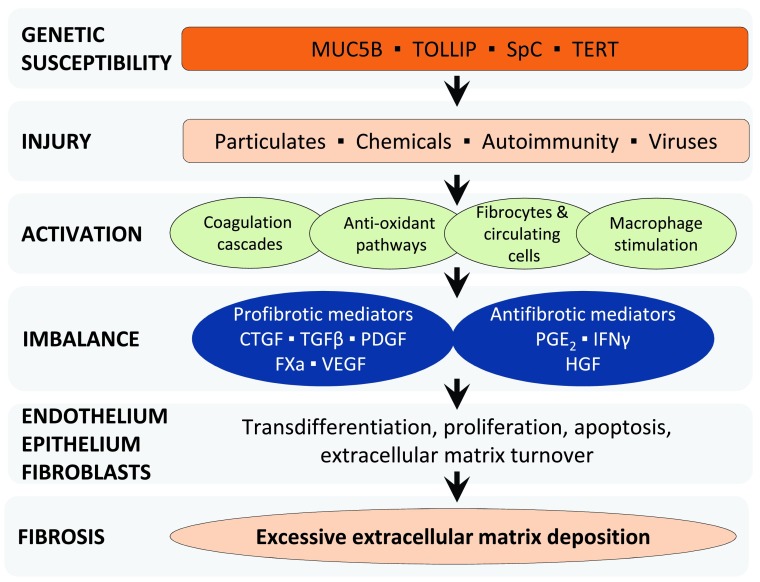
A schematic representing the current model for the pathogenesis of idiopathic pulmonary fibrosis. In genetically susceptible individuals, injury activates multiple inflammatory, cell signalling, and repair pathways. Activation of these cascades causes an imbalance in profibrotic and antifibrotic mediators. In turn, these mediators activate multiple cell types, causing changes in cellular functioning and cell-cell interactions that ultimately result in progressive fibrosis. Abbreviations: CTGF, connective tissue growth factor; FXa, factor Xa; HGF, hepatocyte growth factor; IFNγ, interferon-γ; PDGF, platelet-derived growth factor; PGE2, prostaglandin E2; TGFβ, transforming growth factor β, Th, T-helper; VEGF, vascular endothelial growth factor.

This pathogenic cascade involves complex cell-cell and cell-matrix interactions through numerous biochemical mediators, such as growth factors, enzymes, chemokines, coagulation factors, and reactive oxygen species, all of which have the potential to be influenced by numerous host and environmental factors ^16– 19^. Cardinal among these is transforming growth factor-beta (TGF-β), a potent profibrotic mediator involved in cell recruitment, myofibroblast differentiation, and induction of ECM production ^18– 19^ ( Figure 3).

## Disease stratification and personalised medicine

The early manifestations of IPF are, in the absence of a biopsy, frequently difficult to distinguish from other ILDs. Furthermore, the histological hallmark of IPF, UIP, is found in other disorders and so even when a biopsy is available a diagnosis of IPF can remain in doubt. A further challenge for clinicians is the fact that currently available clinical measures do not allow accurate prediction of subsequent disease behaviour that can range from slowly to rapidly progressive and that, in 5% of cases, is punctuated by episodes of rapid acute deterioration or acute exacerbation ^20^.

These challenges highlight the need for the development and validation of diagnostic markers specific to IPF and prognostic markers of future disease behaviour to guide treatment decisions, including referral for transplant ^21^. The recent approval of pirfenidone and nintedanib and the identification of new potential therapeutic targets have created an urgent need for theragnostic markers, i.e. markers able to assess, ideally at an early stage, therapeutic response to a given drug. Such markers could be used to improve patient selection in clinical trials and also to personalise treatment based on an individual’s risk of progression and treatment response. This in turn would avoid unnecessarily exposing individuals to side effects and would improve the cost-effectiveness of treatment. This approach of disease stratification and personalised medicine is already used in the routine management of cancers and has the potential to improve clinical care in IPF.

## Clinical phenotyping

Several clinical, physiologic, radiographic, and pathologic variables enable a certain degree of mortality prediction in IPF. Older age, male sex, smoking history, low body mass index (BMI), pulmonary hypertension, and concomitant emphysema are clinical predictors of worse survival ^20^. Longitudinal changes in forced vital capacity (FVC) and diffusion capacity for carbon monoxide (DL _CO_) are more predictive of prognosis than baseline values. Thus, a 5–10% decline in FVC at 6 months is associated with a more than twofold increase in the risk of mortality over the subsequent year ^22^. Using relative change in FVC instead of the absolute change enables earlier detection of progression with similar prognostic accuracy ^23^. Also reported as independent predictors of mortality are baseline 6-minute walk distance (6MWD) and change in 6MWD at 6 months ^24^.

Additionally, several multi-dimensional risk prediction models integrating various clinical, physiological, and radiological variables have been validated in IPF ^25– 29^ ( Table 1). These composite staging systems are more accurate in predicting baseline and longitudinal mortality risk than individual physiological variables and permit stratification of IPF patients into groups with distinct patterns of survival. Nevertheless, they cannot reliably predict future disease behaviour (as measured by rate of decline in FVC) or response to treatment ^30^. Additionally, they provide no insights into underlying pathobiology and thus fail to identify distinct molecular phenotypes of disease. The integration of dynamic parameters measured over time and biological biomarkers able to reflect disease activity is needed to improve the accuracy of disease stratification models and guide personalised management ^31^.

**Table 1.** Comparison of mortality risk scoring systems in idiopathic pulmonary fibrosis.

	Variables	Predictive value	Advantages (+)/disadvantages (-)
**Composite** **physiologic index** **(CPI) ^25^**	**DL _CO_**, *% pred* **FVC** *, % pred* **FEV1** *, % pred* *Correlation with morphologic* *extent:* **CPI = 91.0 – (0.65 x DL _CO_)** **– (0.53 x FVC) + (0.34 FEV1)**	More accurate predictor of mortality than individual functional variables	+ : corrects for confounding effects of emphysema - : retrospective data; measurement variability in DL _CO_; not yet been replicated
**du Bois *et al.* model ^26^**	**Age** (0–8 pts) **24-week history of** **respiratory hospitalisation** (0 or 14 pts) **FVC**, *% pred* (0–18 pts) **24-week change in FVC** (0–21 pts)	**1-year mortality risk** *Examples of total score:* • 0–4 pts: > 2% • 22–29 pts: 10–20% • 38–40 pts: 40–50% • > 50 pts: > 80%	+ : easily and reliably evaluable; longitudinal variables - : assessed in cohorts with only mild to moderate physiological impairment at baseline and with exclusion of severe emphysema
**Gender-Age-** **Physiology (GAP)** **model ^27^**	**Gender** (0–1 pts) **Age** (0–2 pts) **FVC**, *% pred* (0–2 pts) **DL _CO_**, *% pred* (0–3 pts)	**Cumulative mortality at** **1, 2, and 3 years** *Examples of 1-year* *mortality risk:* Stage I (0–3 pts): 6% Stage II (4–5 pts): 16% Stage III (6–8 pts): 39%	+ : externally validated; GAP calculator as add-on tool* - : retrospective data; possible referral bias (academic centres); tends to overestimate risk
**Longitudinal GAP** **model ^28^**	**Gender** (0–1 pts) **Age** (0–4 pts) **FVC**, *% pred* (0–15 pts) **24-week relative change** **in FVC** (0–12 pts) **DL _CO_**, *% pred* (0–23 pts) **Respiratory hospitalisation** (last 24 weeks) (0 or 14 pts)	**1- and 2-year mortality** **risk** *Examples of 1-year* *mortality risk:* • 0–10 pts: < 2% • 27–34 pts: 10–20% • 43–45 pts: 40–50% • 55–60 pts: ≥ 80%	+ : longitudinal risk assessment; prospective data - : no external validation
**CT-GAP model ^29^**	**Gender** **Age** **FVC**, *% pred* **Quantitative CT fibrosis** **score**	**Cumulative mortality at** **1, 2, and 3 years** → Accuracy comparable to that of the original GAP model *Examples of 1-year* *mortality risk:* Stage I (0–3 pts): 5% Stage II (4–5 pts): 19% Stage III (6–8 pts): 43%	+ : alternative model when DL _CO_ unmeasurable or not available; externally validated - : retrospective data; requires expertise in quantification of CT disease extent

Abbreviations: DL _CO_, diffusing capacity of carbon monoxide; FVC, forced vital capacity; FEV1, forced expiratory volume in 1 second; CT, computed tomography; % pred, % predicted; pts, points. *GAP calculator for more precise estimation of risk available at www.annals.org

Interestingly, the development and greater accessibility of ^18^F-fluorodeoxyglucose positron emission tomography ( ^18^F-FDG-PET) may provide a novel method for evaluating disease activity in IPF. Areas of established honeycomb fibrosis appear to be highly metabolically active, as shown by increased ^18^F-FDG uptake on PET/CT ^32^. More importantly, increased ^18^F-FDG uptake is also observed in areas of radiologically normal lung parenchyma on HRCT, suggesting that PET/CT may have a higher sensitivity than HRCT in detecting early disease in IPF and may thus represent a potential useful tool in monitoring disease activity and response to treatment ^33^, albeit one which is limited by radiation exposure.

## Molecular phenotyping

High-throughput -omics technologies enable the rapid, accurate, and simultaneous analysis of high numbers of genes, RNA transcripts, proteins, or metabolites. This in turn has facilitated the emergence of systems biology, a multidisciplinary methodology based on integration models aimed at understanding biological systems as a whole, i.e. as a dynamic network of complex interrelated networks extending from the genome to the environment. This contrasts with linear models that have been used in the past to explain the action of individual genes and proteins ^6^. Such multi-scale modelling should permit mapping of the considerable phenotypic heterogeneity of IPF and may enable the identification of specific molecular phenotypes associated with clinical outcomes that could be used to improve diagnosis accuracy and disease stratification ^21^ ( Table 2).

**Table 2.** Candidate molecular biomarkers in idiopathic pulmonary fibrosis.

	Biomarkers	Potential role	Comments	Ref.
**Genetic**	MUC5B promoter SNPs	Predisposition, prognosis	rs35705950 (minor allele): increased susceptibility, improved survival; rs5743890 (minor allele): reduced susceptibility, reduced survival	43– 45
**Genetic**	TOLLIP SNPs	Predisposition, prognosis		42
**Genetic**	SFTPC, SFTPA2	Predisposition		47
**Genetic**	Telomere-related genes (TERT, TERC, DKC1, RTEL1)	Predisposition	Short telomeres in leucocytes associated with reduced survival	47
**Genetic**	Telomere length	Predisposition, prognosis		47, 49
**Transcriptional**	Lung or peripheral blood gene expression profiles	Diagnosis, prognosis	Example: LYCAT mRNA expression in leucocytes correlated with lung function and survival	51– 56
**Epigenetic**	Lung or peripheral blood miRNAs expression profiles	Diagnosis, prognosis, therapeutic targets	Example: Antifibrotic downregulated miRNAs: miR-29, Let-7d; profibrotic upregulated miRNAs: miR-21, miR-154	64– 69
**Blood proteins**	Surfactant proteins (SP-A, SP-D)	Diagnosis, prognosis	Increased levels predictors of worse survival	71, 72
**Blood proteins**	KL-6/MUC1	Diagnosis, prognosis	Increased levels predictors of worse survival and higher risk of AE	73, 82
**Blood proteins**	cCK18	Diagnosis	Higher levels in IPF but no association with disease severity or outcome	34, 35
**Blood proteins**	CCL18	Prognosis	Baseline concentration > 150 ng/ml associated with higher mortality	74
**Blood proteins**	CXCL13	Prognosis	Elevated levels associated with PH, AE, and worse survival	75, 76
**Blood proteins**	Anti-HSP70 IgG	Prognosis	IgG positivity associated with functional decline and worse survival	34, 35
**Blood proteins**	Periostin	Prognosis	Higher levels in IPF and correlation with disease progression	77
**Blood proteins**	Fibulin-1	Diagnosis, prognosis	Elevated levels in IPF and correlation with disease progression	78
**Blood proteins**	MMP-1, MMP-7	Diagnosis, prognosis	Higher levels associated with disease progression and worse survival	79, 80
**Blood proteins**	IL-8, ICAM-1	Prognosis	High concentrations associated with worse survival	80
**Blood proteins**	LOXL2	Prognosis	Higher levels associated with increased risk for disease progression	81
**Blood proteins**	ECM- neoepitopes	Prognosis	Increased concentrations associated with disease progression and rate of increase predictor of survival	40
**BALF proteins**	S100A9 protein	Diagnosis	Significantly higher levels compared to controls and other fibrotic ILDs	86
**Blood cells**	Fibrocytes	Prognosis	Elevated circulating fibrocytes associated with early mortality	83
**Blood cells**	Semaphorin 7a+ Tregs	Prognosis	Increased Sema 7a+ expression on circulating Tregs associated with rapidly progressive IPF	84
**Lung** **microbiome**	Members of *Staphylococcus* and *Streptococcus* genera	Prognosis	Association with disease progression but causal link not established	38
**Lung** **microbiome**	Total bacterial burden	Prognosis	Independent predictor of decline in lung function and mortality but causal link not established	106

Abbreviations: AE, acute exacerbation; BALF, bronchoalveolar lavage fluid; cCK18, caspase-cleaved cytokeratin-18; CCL18, CC-chemokine ligand 18; CXCL13, C-X-C motif chemokine 13; DKC1, dyskeratosis congenital 1 or dyskerin; ECM, extracellular matrix; HSP, heat shock protein; ICAM-1, intercellular adhesion molecule-1; IL-8, interleukin-8; ILDs, interstitial lung diseases; KL-6/MUC1, Krebs von den Lungen-6/Mucin 1; LOXL2, lysyl oxidase-like 2; LYCAT, lysocardiolipin acyltransferase; miRNAs, microRNAs; MMP, matrix metalloproteinases; MUC5B, mucin 5B; PH, pulmonary hypertension; SFTPA2, surfactant protein A2 gene; SFTPC, surfactant protein C gene; RTEL1, regulator of telomere elongation helicase 1; SNPs, single nucleotide polymorphisms; TERC, telomerase RNA component; TERT, telomerase reverse transcriptase; TOLLIP, Toll-interactive protein; Tregs, regulatory T cells.

Ideal molecular biomarkers should reflect key pathological pathways, be easily and accurately measured, have been validated, and offer added value to currently used approaches ^34^. IPF stratification and personalised management based on molecular biomarkers is not yet available in current clinical practice, but recent advances in understanding the complex pathobiology of IPF has identified candidate biomarkers involved in AEC dysfunction, immune dysregulation, ECM remodelling, and fibroproliferation ^35^. A prerequisite for the use of biomarkers in clinical practice is validation in large well-phenotyped cohorts with longitudinal follow up of both clinical and molecular parameters. Among several cohort studies are the COMET (Correlating Outcomes With Biochemical Markers to Estimate Time to progression in Idiopathic Pulmonary Fibrosis) study in the United States ^36– 38^ and the PROFILE (Prospective Observation of Fibrosis in the Lung Clinical Endpoints) study in the United Kingdom ^39, 40^. The latter is the largest prospective cohort study of incident IPF with over 550 patients recruited, all of whom were naïve for antifibrotic therapy at the time of inclusion.

## Genetic phenotyping

Two large genome-wide association studies (GWAS) have identified several common genetic variants associated with susceptibility to IPF and risk of disease progression. The genes identified are involved in host defence, cell-cell adhesion, and DNA repair ^41, 42^. A single nucleotide polymorphism (SNP) in the promoter region of the *MUC5B* gene, encoding a mucin involved in airway host defence ^43^, is significantly associated with sporadic and familial IPF ^44^ and, paradoxically, with improved survival ^45^. This *MUC5B* promoter polymorphism is not associated with lung fibrosis in scleroderma or sarcoidosis and thus appears to be specific to IPF ^46^. Similarly, several SNPs conferring susceptibility to IPF have been identified within the *TOLLIP* locus ^42^. The *TOLLIP* gene encodes for a protein with reduced expression in patients with IPF and that regulates part of the innate immune system mediated by Toll-like receptor and TGF-β signalling pathways. Surprisingly, the minor allele rs5743890 in *TOLLIP* appears to be protective against the development of IPF but when present tends to be associated with increased mortality.

Studies based on familial IPF have identified rare genetic variants in genes encoding surfactant proteins, including surfactant protein C ( *SFTPC*) and A2 ( *SFTPA2*), and in several genes linked to telomere function, such as *TERT* (which encodes for telomerase reverse transcriptase, a component of the telomerase complex responsible for maintaining telomere length ^47^). Short telomere length as well as evidence of lung parenchymal remodelling and epithelial dysfunction have been identified in asymptomatic first-degree relatives of familial IPF patients and may represent the earliest stages of IPF ^48^. Even in the absence of *TERT* polymorphisms, short telomeres in peripheral blood mononuclear cells (PBMCs) or in AECs are also frequently found in IPF patients and portend a poorer prognosis ^47, 49^. This suggests that both genetic variants and environmental factors such as cigarette smoke play a role in telomere shortening.

The biological role of the various genetic variants in the pathogenesis of IPF has yet to be fully determined. Interestingly, an exploratory *post hoc* study conducted in a subgroup of patients participating in a multi-centre randomised control trial of N-acetylcysteine treatment for IPF suggests that genetic polymorphisms may play a role in determining N-acetylcysteine treatment response ^50^. This remains to be confirmed in a prospective clinical trial.

## Transcriptional phenotyping

Whole RNA microarray analysis of lung tissue from patients with different ILDs has identified disease-specific gene expression signatures that permit UIP to be identified from non-UIP samples ^51, 52^. Furthermore, the comparison of lung gene expression profiles of patients with stable or rapidly progressive IPF has identified 134 transcripts sufficiently upregulated or downregulated in the progressive IPF group to distinguish stable from progressive disease ^53^. Similarly, analysis of the peripheral blood transcriptome in IPF has identified genes differentially expressed between IPF patients and healthy controls and also between those with mild and severe disease ^54, 55^. For example, mRNA expression of lysocardiolipin acyltransferase (LYCAT), a cardiolipin-remodelling enzyme, in PBMCs of IPF patients appeared to be strongly correlated with lung function parameters and survival ^56^.

The identification of these diagnostic or prognostic gene expression signatures is a first step towards the development of molecular tests that could be applied to bronchoscopy samples or peripheral blood, thus allowing less invasive approaches to the diagnosis of IPF and earlier identification of individuals at risk of rapid progression.

## Epigenetic and microRNA regulation phenotyping

DNA methylation ^57, 58^, histone modifications ^59, 60^, and noncoding microRNAs (miRNAs) ^61^ are epigenetic mechanisms identified as contributing to differences in gene expression observed in IPF. These regulatory mechanisms are influenced by various factors including environmental exposures (cigarette smoke and infection), genetic profile, sex, and ageing ^62^. A genome-wide DNA methylation analysis of lung tissue identified 2130 significantly differentially methylated regions in IPF samples compared to controls, of which about a third were associated with significant changes in gene expression, including genes identified as IPF-associated common genetic variants ^63^. Thus, dysregulated gene expression in the IPF lung appears to result from complex interactions between genetic and epigenetic factors.

miRNAs influence protein expression by binding to mRNA. Aberrant expression of miRNAs has been described in the pathogenesis of many cancers. Lung tissue miRNA profiling identified significantly increased ^64^ or decreased ^65^ levels of several regulatory miRNAs in IPF patients, thereby distinguishing the normal lung from the IPF lung and rapidly progressive from slowly progressive disease ^66^. TGF-β seems to play a critical role in the upregulation of profibrotic miRNAs and downregulation of antifibrotic miRNAs ^67^. For example, the direct inhibition of let-7d expression by TGF-β in AECs is associated with epithelial to mesenchymal transition and collagen deposition ^68^. Similarly, several circulating miRNAs appear to be differentially expressed in the serum of IPF patients ^67^. Moreover, the expression levels of miR-21, miR-155, and miR-101-3p in serum seem to be correlated with FVC and HRCT features of IPF ^69^. Interestingly, in mice, intravenous injection of synthetic miR-29 during bleomycin-induced pulmonary fibrosis restored endogenous miR-29 function and was followed by decreasing collagen expression and reversal of pulmonary fibrosis ^70^. These changes in miRNA expression in IPF patients suggest that they play an important regulatory role in lung fibrosis and may represent potential diagnostic and prognostic biomarkers as well as therapeutic targets.

## Protein and cell biomarkers

A growing number of studies have sought to identify protein- and cell-based predictors of IPF disease behaviour. Elevated serum levels of several proteins have been associated with worse prognosis in IPF, including surfactant protein A (SP-A) and D (SP-D) ^71, 72^, mucin 1 (KL-6/MUC1) ^73^, CC-chemokine ligand 18 (CCL18) ^74^, C-X-C motif chemokine 13 (CXCL13) ^75, 76^, periostin ^77^, fibulin-1 ^78^, matrix metalloproteinases MMP-1 and MMP-7 ^79, 80^, interleukin-8 (IL-8), intercellular adhesion molecule (ICAM)-1 ^80^, and lysyl oxidase-like 2 protein (LOXL2) ^81^. Elevated baseline serum levels of KL-6/MUC1 also appear to predict the risk of future acute exacerbation ^82^. Similarly, some circulating cells have been associated with worse survival. Among cellular markers of rapidly progressive IPF are elevated circulating fibrocytes ^83^ and semaphorin 7a+ regulatory T cells (Tregs) ^84^.

Serial measurements of serum ECM protein fragments generated by MMP activity in 189 IPF patients recruited in the PROFILE cohort identified increased serum concentrations of these protein fragments in IPF patients compared to controls. More importantly, increasing neoepitope concentrations were associated with disease progression, and the rate of change over 3 months of 3 of these MMP-degraded ECM proteins predicted survival ^40^. These results suggest that serial longitudinal measurement of circulating proteins have potential for use as prognostic or theragnostic biomarkers.

Studies based on lung tissue or bronchoalveolar lavage fluid (BALF) analysis have also identified some candidate diagnostic and prognostic biomarkers of IPF, including αvβ6 integrin ^85^, S100A9 protein ^86^, and soluble annexin V ^87^.

The value of these protein or cell biomarkers as diagnostic or prognostic factors in IPF needs to be further assessed. Furthermore, integrating validated molecular variables in multivariate risk prediction models could improve their accuracy in predicting outcomes in IPF. In view of this, Richards and colleagues formulated the personal clinical and molecular index (PCMI), integrating sex, FVC % predicted, DL _CO_ % predicted, and MMP-7 serum concentration, which accurately predicted mortality in their validation cohort ^80^. Two other prediction models integrating SP-A and SP-D levels or MMP-7, SP-A, and KL-6/MUC1 levels have shown improved predictability of mortality compared with clinical predictors alone ^71, 88^.

## Metabolic phenotyping

Metabolomics is the systematic analysis of the complete set of metabolites (the metabolome) within a biological system under given conditions. This approach offers the potential for a better understanding of dysregulated metabolic pathways underlying numerous diseases, including airway diseases such as asthma, chronic obstructive pulmonary disease (COPD), and cystic fibrosis ^89^. Dysregulated metabolic mechanisms have also been highlighted in the pathogenesis of IPF. Increased levels of lactic acid in IPF lung tissue compared with controls appear to play a role in myofibroblast differentiation via a pH-dependent activation of TGF-β ^90^. Recently, a metabolomic assay by Xie and colleagues demonstrated that augmented aerobic glycolysis, mediated by upregulated glycolytic enzymes, including PFKFB3, represented an early and sustained event during myofibroblast differentiation ^91^. More importantly, PFKFB3 inhibition mitigated myofibroblast differentiation and dampened the profibrotic phenotypes of myofibroblasts isolated from IPF lungs. These data suggest that glycolytic reprogramming is important in the pathogenesis of lung fibrosis and therefore represents a potential therapeutic target. More research is needed in the field of metabolomics to clarify the role of these dysregulated pathways of cellular metabolism in the pathogenesis of IPF and to integrate them with available genetic, epigenetic, transcriptomic, and proteomic data.

## Environmental and host factors

Smoking history has long been described as a prevalent risk factor for the development of IPF ^92^, including familial IPF ^93^, and is associated with a worse survival ^94^. Some other environmental and occupational exposures, including wood, mineral, and metal dusts, agriculture, and livestock, have also been associated with IPF, although a formal causal link has not been established ^95^. Furthermore, air pollution may also play a role in the pathogenesis of IPF. A recent study reported a significantly higher risk of acute exacerbation of IPF with increased ozone and nitrogen dioxide exposure over the preceding 6 weeks ^96^.

Gastroesophageal reflux (GER) is highly prevalent in IPF, though often asymptomatic, and confers an increased risk of microaspiration ^97^. Anti-acid treatment in IPF has been associated in retrospective data with decreased radiologic fibrosis, longer survival, and smaller decrease of FVC at 30 weeks ^98, 99^. Despite growing evidence suggesting that GER and silent microaspiration might play a role in the pathogenesis of IPF, there is, to date, no confirmation that this association is causative. Consequently, the recently updated international guidelines on IPF treatment maintained a conditional recommendation for the use of anti-acid therapy ^100^. A prospective randomised controlled trial is needed to further assess the role of GER and microaspiration in IPF and confirm the effectiveness of anti-reflux therapy. 

Infectious processes may play a role in the initiation, progression, or exacerbation of IPF. Viral infections, particularly human herpes viruses (HHVs), including herpes simplex virus type 1 (HSV-1), Epstein-Barr virus (EBV), cytomegalovirus (CMV), HHV-7, and HHV-8, have been associated with IPF in several studies ^101^. Whether this association is causative has not yet been proven. HHVs have the potential to induce endoplasmic reticulum stress and apoptosis ^102^; it is therefore hypothesised that viral infection may act as a cofactor in the development of IPF through the reactivation of latent HHVs within the alveolar epithelium following exposure to a first injury ^103^. Furthermore, a recent study found increased copy numbers of EBV and CMV DNA in BALF of IPF patients and, to a lesser extent, in first-degree asymptomatic relatives of familial IPF patients ^48^. Thus, enhanced HHV replication may trigger epithelial cell stress and participate in disease initiation. A small clinical trial of ganciclovir in individuals with severe IPF with positive EBV-IgG serology showed a modest improvement in surrogate markers of disease progression ^104^. It has recently been reported that influenza infection may also play a role in lung fibrosis by promoting collagen deposition via αvβ6 integrin-mediated TGF-β activation in epithelial cells ^105^.

Recent data also suggest a putative role for bacteria and lung microbiome in IPF. An analysis of the COMET study showed an association between progression of IPF and the presence of specific members within the *Staphylococcus* and *Streptococcus* genera in BALF ^38^. Similarly, Molyneaux and colleagues found an increased bacterial load, consisting particularly of *Haemophilus*, *Streptococcus*, *Neisseria*, and *Veillonella* spp., in BALF of IPF patients compared to healthy smokers, nonsmokers, and patients with moderate COPD ^106^. More importantly, the total bacterial burden was an independent predictor of decline in lung function and mortality. Whether these differences in lung microbiome are a cause or consequence of IPF is unknown. A clinical trial of 12 months of co-trimoxazole in addition to standard treatment in 181 patients with fibrotic idiopathic interstitial pneumonia (about 90% of whom had IPF) showed a reduction in mortality but did not slow functional decline ^107^. The exact role of viruses and bacteria in the pathogenesis of IPF has yet to be determined and the potential for antiviral or antibiotic treatments requires further evaluation.

## Conclusion

Currently available therapies for IPF are of limited efficacy, and the prognosis associated with the condition remains poor. Recent advances in our understanding of the complex interrelated mechanisms underlying fibrosis in the lung are encouraging and pave the way towards an integrated approach to diagnosis, stratification, and treatment. It is becoming increasingly clear that genetic polymorphisms, whole blood transcriptomic profile, and lavage microbiome all predict groups of patients with differing disease behaviour and outcomes and potentially variable responses to treatment. Furthermore, prospective longitudinal cohort studies have started to identify blood biomarkers that have the potential to be used as early measures of treatment response. Considerable further research is required to deliver personalised medicine for IPF into the clinic, but at least now there is light at the end of what has been a very long tunnel.
